# *In Vivo* and* In Vitro* Toxicity Evaluation of Hydroethanolic Extract of* Kalanchoe brasiliensis* (Crassulaceae) Leaves

**DOI:** 10.1155/2018/6849765

**Published:** 2018-01-21

**Authors:** Aldilane Gonçalves Fonseca, Luzia Leiros Sena Fernandes Ribeiro Dantas, Júlia Morais Fernandes, Silvana Maria Zucolotto, Adley Antoninni Neves Lima, Luiz Alberto Lira Soares, Hugo Alexandre Oliveira Rocha, Telma Maria Araújo Moura Lemos

**Affiliations:** ^1^Department of Clinical and Toxicological Analysis, Federal University of Rio Grande do Norte, Natal, RN, Brazil; ^2^Department of Pharmacy, Federal University of Rio Grande do Norte, Natal, RN, Brazil; ^3^Department of Pharmacy, Federal University of Pernambuco, Recife, PE, Brazil; ^4^Department of Biochemistry, Federal University of Rio Grande do Norte, Natal, RN, Brazil

## Abstract

The species* Kalanchoe brasiliensis*, known as* “Saião*,*”* has anti-inflammatory, antimicrobial, and antihistamine activities. It also has the quercetin and kaempferol flavonoids, which exert their therapeutic activities. With extensive popular use besides the defined therapeutical properties, the study of possible side effects is indispensable. The objective of this study is to evaluate the toxicity* in vitro* and* in vivo* from the hydroethanolic extract of the leaves of* K. brasiliensis*. The action of the extract (concentrations from 0.1 to 1000 uL/100 uL) in normal and tumor cells was evaluated using the MTT method. Acute toxicity and subchronic toxicity were evaluated in mice with doses of 250 to 1000 mg/kg orally, following recognized protocols. The* in vitro* results indicated cytotoxic activity for 3T3 cell line (normal) and 786-0 (kidney carcinoma), showing the activity to be concentration-dependent, reaching 92.23% cell inhibition.* In vivo*, the extract showed no significant toxicity; only liver changes related to acute toxicity and some signs of liver damage, combining biochemical and histological data. In general, the extract showed low or no toxicity, introducing itself as safe for use with promising therapeutic potential.

## 1. Introduction

The genus* Kalanchoe* is one of the most important genera of the family Crassulaceae used in traditional medicine and it comprises approximately 125 species. Among these species,* Kalanchoe brasiliensis* Cambess stands out, popularly known as* “Saião”* and* “coirama*,*”* and is widely used in traditional medicine for the treatment of cough, erysipelas, and others due to its anti-inflammatory action [1]. The species is native to Brazil, being common from São Paulo to the Northeast, mainly in the coastal zone [1].

Regarding the chemical constitution, the main components described to* Kalanchoe brasiliensis *leaf are glycosylated flavonoids derived from aglycone patuletin and eupafolin [2]

Studies described antiproliferative and antihistaminic activities, inhibition of thyroid peroxidase, antiviral activities, anticholinesterase, larvicide, and antimicrobial and mainly anti-inflammatory activities [1–8].

The toxicity of the* K. brasiliensis* species has been poorly studied over the years and therefore its importance in folk medicine and the scarcity of studies confirming its safe use have revealed the need to carry out toxicity studies of* K. brasiliensis*. Thus, the objective of the present work was to evaluate the* in vivo* and* in vitro* toxicity of the hydroethanolic extract of the leaves of* Kalanchoe brasiliensis* (Crassulaceae).

## 2. Materials and Methods

### 2.1. Plant Material

Leaves of* Kalanchoe brasiliensis* were collected in the city of Macaíba (coordinates: 5°51′30′′S 35°21′14′′W), Rio Grande do Norte, Brazil, in September 2010. The botanical identification was performed by Dr. Maria Iracema Bezerra Loyola and a voucher was deposited in the Herbarium of the Center of Biosciences of the Federal University of Rio Grande do Norte, Brazil (UFRN 5468). The collection of the plant material was carried out under authorization of the Brazilian System of Information on Biodiversity (SISBIO) (process number: 35017).

The extract was prepared and analyzed according to the methodology described by Costa et al. (2015) and Fernandes et al. (2016) and assigned by Professor Dr. Silvana Maria Zucolotto from the Pharmacognosy Laboratory of UFRN. For the evaluation of* in vivo* toxicity, the extract was diluted in 0.9% physiological solution and for* in vitro* toxicity, the extract was diluted in the culture medium.

### 2.2. Animals

The experimental protocols were approved by the Animal Ethics Committee of the Federal University of Rio Grande do Norte (Protocol 002/2013). Male and female* Swiss *mice* (Mus musculus)*, aged 6–8 weeks, weighing 30 ± 2 g, were kept at the UFRN Health Sciences Center vivarium in controlled illumination (12-hour light/dark cycle) and temperature (23 ± 2°C) conditions. They received water and commercial food ad libitum.

### 2.3. Acute Toxicological Assessment

Randomly, 48 animals were separated (*n* = 6/sex/group) in experimental groups receiving oral doses of 250, 500, and 1000 mg/kg and a control group received saline solution through an appropriate cannula in single dose [9–11].

The mice were observed during the first 24 hours for the beginning of any immediate toxic signs and daily, during 14 days, to observe death, behavioral changes, and any acute delayed effect. The body weight and the consumption of food and water were measured on alternate days. After 14 days, the animals were anesthetized with thiopental, 40 mg/kg (Tiopentax®, Cristália), intraperitoneally (ip), and submitted to euthanasia by cervical dislocation, according to the CONCEA Regulations 2016 [12].

### 2.4. Subchronic Toxicological Assessment

40 animals were randomized (*n* = 5/sex/group) into experimental groups which received oral doses of 250, 500, and 1000 mg/kg for 30 days and a control group receiving saline solution through a proper cannula in a single daily dose [10, 11, 13]. Mice were observed during the first 24 hours for the beginning of any immediate toxic signs and daily for 30 days. The body weight and the consumption of food and water were measured on alternate days. After 30 days, the animals were anesthetized with thiopental, 40 mg/kg (Tiopentax, Cristália, Brazil), intraperitoneally (ip), and submitted to euthanasia by cervical dislocation, according to the CONCEA Regulations [12].

### 2.5. Biochemical and Hematological Determinations

Blood samples were obtained by cardiac puncture and collected without anticoagulant for biochemical dosages and with anticoagulant (EDTA) for complete blood count. The biochemical parameters analyzed from serum were glucose (G), total cholesterol (TC), triglycerides (TG), aspartate aminotransferase (AST), alanine aminotransferase (ALT), urea (Ur), creatinine (Cr), and total protein (TP), performed with appropriate Labmax Plenno automatic biochemical analyzer diagnostic kits (Labtest, Brazil). The hematological parameters analyzed were white blood cells (WBC) and differential count of leukocytes, platelets, hematocrit and hemoglobin (Hb), mean corpuscular volume (MCV), mean corpuscular hemoglobin (MCH), and mean corpuscular hemoglobin concentration (MCHC), performed by automatic analyzer ABX Micros 60-OT (Horiba ABX, Japan).

### 2.6. Histopathological Analysis

For histopathological analysis, fragments of collected organs (kidney, liver, and spleen) were examined macroscopically and weighed and the organ/total body weight ratio was calculated. The fragments were fixed in 10% formaldehyde and referred to the Pathology Department of Federal University of Rio Grande do Norte for histological analysis by Professor Cláudia Nunes Oliveira. They were stained with hematoxylin and eosin and tissue injury parameters were investigated through presence or absence of apoptosis, necrosis, hydropic degeneration, steatosis, cholestasis, ductile proliferation, fibrosis, inflammatory response, tubular swelling, and edema [14].

### 2.7. Cell Culture

Cells lines from nontumoral mouse fibroblasts (3T3) and renal carcinoma (786-0) mice fibroblast cell lines were donated by Professor Dr. Hugo Alexandre de Oliveira Rocha of the Department of Biochemistry of Federal University of Rio Grande do Norte, acquired from the American Type Culture Colletion (ATCC, Rockville, MD, USA). They were maintained in Dulbecco's Modified Eagle's medium (DMEM) (Sigma-Aldrich, Co., Germany) and supplemented with 10% FBS, at the concentration of 2,5 × 10^4^/mL, in 10 mL medium, in a 75 cm^3^ culture bottle. Cells were picked every two days, using 0.25% trypsin in PBS, centrifuged, and resuspended in fresh culture medium.

### 2.8. Cell Viability/MTT Assay (3-(4,5-Dimethylthiazol-2-yl)-2,5-diphenyltetrazolium bromide)

The cell viability of the hydroalcoholic extract was analyzed in cell cultures at different concentrations and time periods. Cells were plated in 96-well microplates at the concentration of 5 × 10^4^ cell/well. They were plated in DMEM medium supplemented with 10% FBS for cell maintenance and growth and maintained for 24 hours in an incubator at 36.5°C with 5% of CO^2^. Subsequently, a plate was deprived and left for 24 hours under the same conditions. After removal of the supernatant, concentrations of 1, 10, 10^2^, 10^3^, and 10^4^ *μ*L/mL of the extract in DMEM medium (10% FBS) were added in triplicate to the plate. After 24-, 48-, and 72-hour incubation, the entire media content in the microplates was discarded; the 10% MTT reagent (Oz Biosciences, San Diego, USA) was added to the wells. After 4 hours, the supernatant was discarded, and 100 *μ*L of absolute ethanol was added for solubilization of the crystals formed. The absorbance of each well was measured in Elisa Reader 570 nm [15, 16].

### 2.9. Statistical Analysis

Statistical analysis was performed using SPSS v.20 for Windows (SPSS, Chicago, IL, USA). For independent samples, ANOVA was applied with Tukey-Kramer posttest. The nonparametric variables were analyzed by the Kruskal-Wallis test. Histopathological analyses were evaluated by Fisher's test. All tests were calculated at a significance level of 5%.

## 3. Results and Discussion

### 3.1. Assessment of Acute and Subchronic Toxicity

In the acute evaluation, the biochemical parameters of glucose, triglycerides, ALT, and creatinine showed a significant difference (*p* < 0.05) between the groups of animals (Table 1). In the subchronic evaluation, the parameters glucose, AST, and creatinine presented significant difference (*p* < 0.05) between the groups (Table 2).

In the hematoxicity tests, there was no statistically significant difference between the groups; however, the male group at the 1000 mg/kg dose presented high granulocytes in relation to the control group (*p* < 0.05) and there were alterations between males and females of the control groups for hematocrit, platelets, lymphocytes, and monocytes.

### 3.2. Histopathological Analysis

Histopathological analysis revealed unchanged findings in most animals. Hepatic steatosis (EH), inflammatory reaction (RI), extra spinal hematopoiesis (ESH), hepatic congestion (CH), and renal congestion (CR) showed no difference between any of the groups.

The pancreas presented no histological changes in any of the animals in the acute and subchronic evaluation, as well as for the renal histopathology in the acute evaluation. However, in the subchronic evaluation, cellular infiltrate in the kidney was evidenced in the females of the 500 and 1000 mg/kg groups.

**Figure 1:**
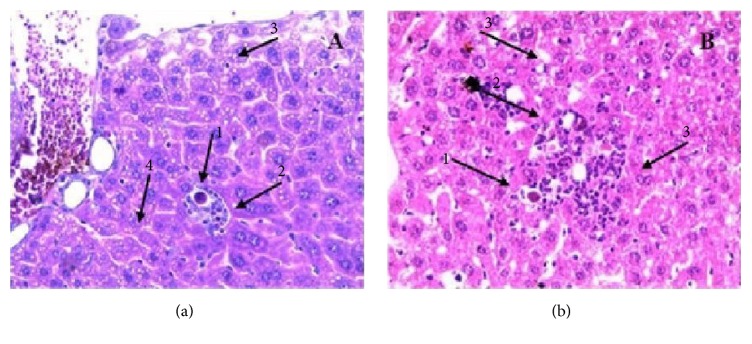
Liver tissue presenting apoptosis (1), extramedullary hematopoiesis (2), hepatic steatosis (3), and congestion (4) in animals subject to a dose of 500 mg/kg of* K. brasiliensis* in acute toxicity (A) and hepatic tissue presenting apoptosis (1), extramedullary hematopoiesis (2), and hydropic and hepatic degeneration (3) in the animal subject to a dose of 1000 mg/kg of the extract of* K. brasiliensis* in the acute toxicity evaluation (B) (10x).

**Figure 2:**
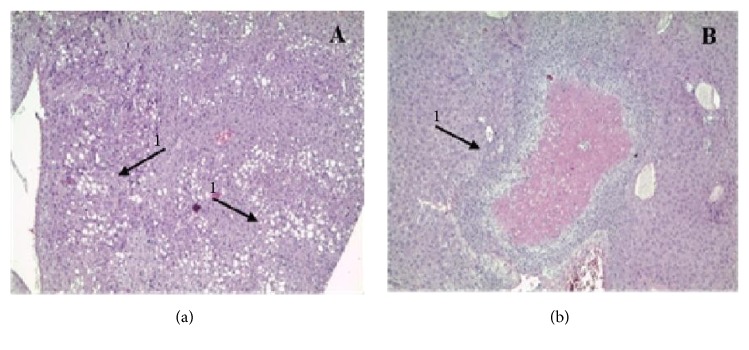
Liver tissue with presence of moderate steatosis (1) in the animal subject to a dose of 1000 mg/kg of* K. brasiliensis* extract in the subchronic evaluation (10x) (A). Ischemic renal tissue with coagulative necrosis (1) in the animal submitted to a dose of 1000 mg/kg of* K. brasiliensis* extract in the subchronic evaluation (10x) (B).

Apoptosis was present in the dose groups of 500 mg/kg and 1000 mg/kg in the acute study (Figure 1). Subchronic evaluation showed moderate steatosis (Figure 2(a)) and punctual ischemic necrosis in the 1000 mg/kg dose group (Figure 2(b)).

### 3.3. Cell Viability Assessment

**Figure 3:**
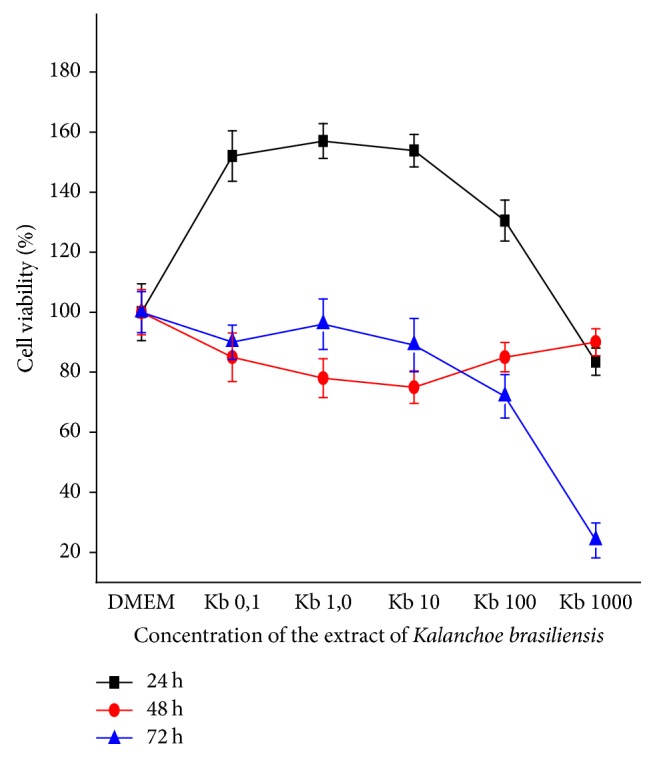
Viability of nontumoral cells (3T3) at periods of 24-, 48-, and 72-hour exposure to concentrations of 1–10^4^ *μ*L/mL of* K. brasiliensis* extract by the MTT method.

In the 24-hour period in the 3T3 strain, the extract showed cell viability at concentrations of 10 to 10^3^ *μ*g/mL and impairment of cell viability at concentrations of 1 and 10^4^ *μ*g/mL up to 80%, As well as in 48 hours at concentrations of 1 to 10^2^ *μ*g/mL, ranging from 10,34 to 25,74%. The concentrations of 10^3^ and 10^4^ *μ*g/mL presented cell viability of 93,99%; and at 72 hours, all concentrations caused impairment in cell viability, ranging from 10,53 to 79,81% (Figure 3).

**Figure 4:**
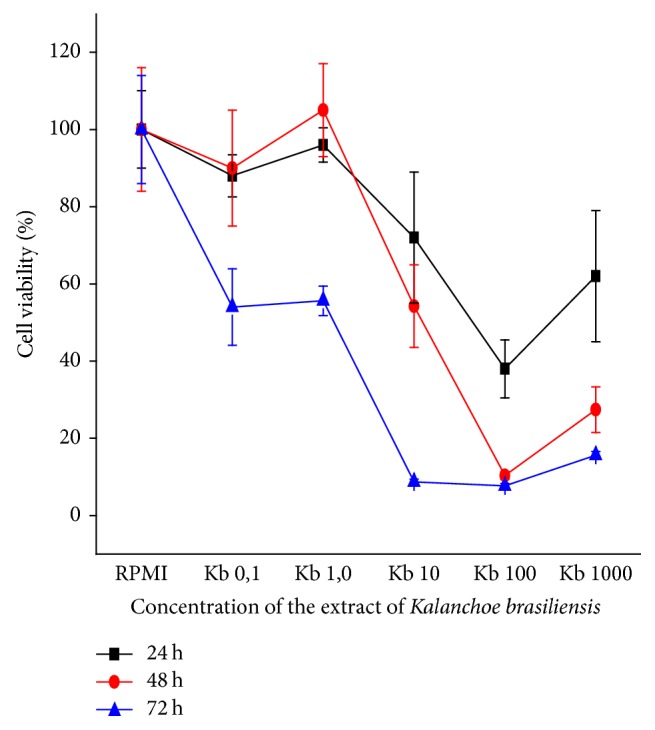
Viability of renal carcinoma cells (786-0) at periods of 24-, 48-, and 72-hour exposure to concentrations of 1–10^4^ *μ*L/mL of* K. brasiliensis* extract by the MTT method.

The 786-0 cells, in the 24-hour period, had the cellular viability compromised at all concentrations, with 10^3^ *μ*g/mL impaired in 61,68%; in 48 hours, there was also this compromise of cellular viability in 89,65% and 72,57% in the concentrations of 10^3^ and 10^4^ *μ*g/mL, respectively, as well as in 72 hours, in the concentrations of 10^2^, 10^3^, and 10^4^ *μ*g/mL, and there was impairment of cell viability of 91,59%, 92,26%, and 84,44%, respectively (Figure 4).

Body weight is one of the parameters most used in toxicological evaluations to indicate the appearance of toxic effects of a certain substance in the animal organism, as well as the reduction in water consumption and feed intake, behavioral alteration, apathy, and bad hair condition. The absence of these signals between the groups of animals suggests that the extract does not present toxicity under these conditions. Studies in the literature corroborate these results [17, 18].

The biochemical parameters metabolically reflect the physiological status of the animals submitted to the toxicity study. Glucose can be altered by several factors such as diet and intestinal absorption [19]. The alterations found in glucose for acute evaluation (dose 250 mg/kg) and subchronic evaluation (females dose 1000 mg/kg) do not determine the development of diabetes in the animals, as there may be competition of the glucose transporter with the glycosylated quercetin, interfering in the concentration of serum glucose, or even stress that the animal has suffered [20]. Kaempferol, also found in the extract, stimulates the uptake of glucose in cells of normal animals, but these flavonoids need the presence of sugars linked to the flavonoid functional nucleus to initiate their insulinomimetic action [21, 22].

However, the portion of sugar bound to the flavonoids increases the polarity of the molecule and causes a steric hindrance to bind with the carrier proteins in the bloodstream; thus, more glycosylated portion is free, being responsible for the glucose increase in concentration in the toxicity evaluation. Furthermore, the initial overload that the liver may have suffered to metabolize the extract may have influenced the glucose concentration. And this overload is no longer felt in the subchronic evaluation [23].

A possible explanation for the discrepancies among the results in this study and literature might be related to the choice of route of administration, dose, extract type, and the animal species in which the extracts were tested.

In both acute and subchronic evaluations, the triglycerides profiles were similar, with smaller mean triglycerides with increasing dose, with a difference in the acute evaluation (dose 1000 mg/kg) and subchronic evaluation (female dose 250 mg/kg). It is suggested that the extract shows activity on the triglycerides, but the mechanism of action cannot be described; it is only supposed that the inhibitory action of enzymes of this metabolism or the increase of the antioxidant activity of the flavonoids present in this plant extract is responsible for the activity [24].

Total cholesterol did not differ between groups. Studies of plants with hypocholesterolemic effects correlate this effect to the inhibition of enzymes involved in lipid metabolism by action, mainly of flavonoids, which act with antioxidant effect through the reduction of lipoperoxidation and increase of antioxidant enzymes activity [25, 26].

Liver enzymes aspartate aminotransferase (AST) and alanine aminotransferase (ALT) evaluated hepatic injury. The higher activity of ALT in the 500 and 1000 mg/kg dose groups in females and 500 mg/kg in males in the acute assay suggests hepatic damage, highlighting the acute toxicity at these doses [24, 26, 27]. Females are more susceptible to changes, which can be better understood with histopathological findings. The alteration of the AST (male dose 1000 mg/kg), in the subchronic evaluation, suggests that the extract caused some deeper lesion in the organ, visualized in the histopathological analysis.

Synthesized by the liver, the total protein reflects liver functional capacity, which did not demonstrate hepatocellular toxic effect through this dosage. The dosages of AST, ALT, and total proteins demonstrate the hepatic profile evaluated in lesion and function, and it is possible to affirm that the extract of* K. brasiliensis* does not promote any severe hepatic alteration, since the synthesis function of the organ remains intact, and the alteration in ALT liver enzyme demonstrates that the damage may be reversible [24, 27]. Renal function, analyzed through the dosages of urea and creatinine, showed no change corresponding to toxicity in the acute and subchronic evaluation. However, in the acute evaluation (females dose 1000 mg/kg) and subchronic evaluation (females dose 500 mg/kg), there were some differences in urea which may be associated with the histological findings, and therefore it is suggested that the extract did not cause any renal damage by the presence of protective compounds such as quercetin, ensuring the safety of the extract.

On the other hand, creatinine (female dose 1000 mg/kg) presented a difference and may have occurred due to the action of female hormones or muscle mass inferior to males and its effect was not attributed to the extract.

It is also suggested that changes in hematological parameters are not attributed to the effects caused by the extract, since they are expected changes for the species [28, 29]. Some studies have shown that acute induced stress in animals causes the release of catecholamines and recruitment of leukocytes, which induce the increase of cells in the circulation, which was observed in the subchronic toxicity evaluation in the highest dose of the extract [30].

In the histopathology, the findings of cellular infiltration in the kidneys, in the subchronic evaluation, characteristic of inflammation, have an ascending and pelvic trait, and therefore it is not attributed to the extract, since the alteration occurs in females that are more susceptible to infection in the urinary tract [31]. The acute apoptosis (doses of 500 and 1000 mg/kg) (Figure 1) deserves attention for these doses, as well as the presence of punctual ischemic necrosis (Figure 2(b)) in the subchronic evaluation (dose of 1000 mg/kg), suggesting that this effect of cell damage is attributed to the extract.

Studies corroborate the safety of the extract due to the maintenance of renal histological integrity, attributing this effect to the quercetin [20, 32]. As for hepatic histology, it is suggested that the extract caused acute and mild hepatocellular damage, corroborating with the biochemical data found [19, 24, 25, 32].

There is a need to find new substances capable of differentiating their action between normal and cancerous cells. This is a necessary criterion for selectively killing cancerous cells and selective natural products have become highly needed [33]. In this study, in the evaluation of cell viability, normal cells (3T3) were not impaired with the extract, whereas for 786-0 lineage, there was considerable impairment. These results highlight the importance of in-depth studies for this species with promising therapeutic activity.

Previous work [2, 3] in our group reported a presence of glycosylated flavonoids with aglycones derived mainly from patuletin and eupafolin. According to these results, it is possible to hypothesize that flavonoids can be involved with activity of* K. brasiliensis* extract in 786-0 cells, since they act in the control of free radical-mediated pathogenesis through the reduction of alkylperoxyl radicals (ROO^−^) [34]. Studies have found that phenolic compounds may act in the prevention of cancer, as well as in inhibition of cell cycle differentiation, inducing apoptosis [34–36]. The prooxidant effect of quercetin may also be responsible for the cytotoxic and proapoptotic effects [20]. Moreover, Berger et al.'s (2013) studies show that kaempferol acts on the inhibition of histone deacetylases, which are associated with the development of neoplasias, suggesting that the extract is promising for the development of anticancer drugs [37].

One of the limitations of the present study is that the toxicity of none of the fractions of this extract has not yet been investigated and also that the animals are very sensitive to the stress and this can reflect on the observed parameters. However, this disadvantage does not exclude the results found by this work.

## 4. Conclusions

*Kalanchoe brasiliensis* leaf extract impaired cell viability in the 786-0 cell line, especially at higher doses with a similar cellular inhibition profile at all observed times. This study also demonstrated that the extract has a low acute* in vivo* toxic effect, most evident at the dose of 1000 mg/kg observed in ALT concentrations and in the liver histopathology. And regarding subchronic toxicity, through histological findings, low relative toxicity is concluded. The extract of the leaves of* Kalanchoe brasiliensis* presents itself as a promising object of study for the development of herbal drugs. However, further studies are still needed about its toxicity to ensure its safety.

## Figures and Tables

**Table 1:** Results of the biochemical dosages in the evaluation of acute toxicity of *K. brasiliensis*.

Parameters/groups	Control	250 mg/kg	500 mg/kg	1000 mg/kg
	Males			
Glucose (mg/dl)	102,30 ± 95,39	202,00 ± 75,16^*∗*^	215,80 ± 56,44	190,60 ± 67,77^*∗*^
Triglycerides (mg/dl)	94,11 ± 41,97	81,20 ± 20,85	78,20 ± 14,57	73,20 ± 14,24^*∗*^
Cholesterol (mg/dl)	111,89 ± 26,54	202,60 ± 64,10	114,40 ± 45,77	116,40 ± 15,34
TP (mg/dl)	4,19 ± 0,85	2,16 ± 1,69	5,08 ± 1,57	4,64 ± 0,83
AST (U/L)	131,22 ± 56,82	148,60 ± 31,11	148,00 ± 18,55^*∗*^	152,40 ± 56,29
ALT (U/L)	56,70 ± 15,88	55,40 ± 13,22	70,80 ± 7,60^*∗*^	46,80 ± 15,47
Urea (mg/dl)	72,56 ± 6,06	82,20 ± 12,26	70,60 ± 8,08^*∗*^	76,00 ± 6,96
Creatinine (mg/dl)	0,13 ± 0,20	0,48 ± 0,33^*∗*^	0,24 ± 0,22	0,40 ± 0,00^*∗*^

	Females			
Glucose (mg/dl)	144,20 ± 50,25	226,20 ± 53,27^*∗*^	126,80 ± 41,14	234,60 ± 38,60^*∗*^
Triglycerides (mg/dl)	114,40 ± 31,09	144,60 ± 44,50	81,40 ± 18,15	72,00 ± 8,19^*∗*^
Cholesterol (mg/dl)	124,40 ± 11,67	106,60 ± 19,19	75,00 ± 14,88	102,20 ± 10,85
TP (mg/dl)	2,92 ± 0,85	5,46 ± 0,55	3,76 ± 0,91	3,64 ± 0,46
AST (U/L)	151,80 ± 82,79	201,60 ± 195,69	127,40 ± 6,88^*∗*^	147,20 ± 57,84^*∗*^
ALT (U/L)	70,40 ± 2,07	53,00 ± 14,76	72,20 ± 5,76	38,20 ± 12,05
Urea (mg/dl)	73,60 ± 2,07	59,00 ± 7,91	53,20 ± 7,79^*∗*^	54,80 ± 11,88
Creatinine (mg/dl)	0,16 ± 0,22	0,42 ± 0,04^*∗*^	0,24 ± 0,22	0,22 ± 0,16^*∗*^

Values are expressed as mean ± standard deviation (*n* = 6). AST: aspartate aminotransferase; ALT: alanine aminotransferase; TP: total protein.  ^*∗*^Groups presenting statistical difference with the control group (*p* < 0.05).

**Table 2:** Results of the biochemical dosages in the evaluation of subchronic toxicity of *K. brasiliensis*.

Parameters/groups	Control	250 mg/kg	500 mg/kg	1000 mg/kg
	Males			
Glucose (mg/dl)	56,375 ± 34,912	99,60 ± 23,787	93,00 ± 34,169	82,40 ± 22,534
Triglycerides (mg/dl)	109,80 ± 48,634	92,60 ± 22,974	92,20 ± 26,243	85,800 ± 38,311
Cholesterol (mg/dl)	70,10 ± 25,632	64,40 ± 22,244	82,20 ± 35,160	80,600 ± 16,273
TP (mg/dl)	4,556 ± 1,014	4,20 ± 0,8367	4,60 ± 0,5477	4,60 ± 1,140
AST (U/L)	120,30 ± 40,415	122,60 ± 26,81	214,20 ± 106,00	216,80 ± 113,7^*∗*^
ALT (U/L)	52,00 ± 26,051	37,40 ± 5,459	85,20 ± 40,69	85,60 ± 69,20
Urea (mg/dl)	51,80 ± 9,426	45,20 ± 11,713	42,20 ± 1,789	62,40 ± 8,877
Creatinine (mg/dl)	0,508 ± 0,0702	0,548 ± 0,08402	0,600 ± 0,0	0,694 ± 0,1915

	Females			
Glucose (mg/dl)	64,375 ± 30,863	97,80 ± 27,087	96,20 ± 37,265	123,80 ± 20,41^*∗*^
Triglycerides (mg/dl)	75,875 ± 22,10	152,20 ± 20,19^*∗*^	151,25 ± 98,215	94,00 ± 66,142
Cholesterol (mg/dl)	67,00 ± 37,74	80,40 ± 47,479	53,750 ± 17,84	64,40 ± 23,522
TP (mg/dl)	3,50 ± 0,9718	5,00 ± 0,7071	4,60 ± 0,8944	4,60 ± 0,1342
AST (U/L)	159,67 ± 84,714	130,60 ± 58,188	116,00 ± 6,519	156,00 ± 99,82^*∗*^
ALT (U/L)	65,11 ± 58,91	39,60 ± 5,030	45,00 ± 5,244	69,20 ± 31,83
Urea (mg/dl)	48,30 ± 9,31	47,00 ± 6,745	43,80 ± 6,907	49,20 ± 12,637
Creatinine (mg/dl)	0,7355 ± 0,4784	0,5975 ± 0,0689	0,925 ± 0,0957^*∗*^	0,600 ± 0,00

Values are expressed as mean ± standard deviation (*n* = 5). AST: aspartate aminotransferase; ALT: alanine aminotransferase; TP: total protein.  ^*∗*^Groups presenting statistical difference with the control group (*p* < 0.05).
